# Sleep-Aware Adaptive Deep Brain Stimulation Control: Chronic Use at Home With Dual Independent Linear Discriminate Detectors

**DOI:** 10.3389/fnins.2021.732499

**Published:** 2021-10-18

**Authors:** Ro’ee Gilron, Simon Little, Robert Wilt, Randy Perrone, Juan Anso, Philip A. Starr

**Affiliations:** ^1^Department of Neurological Surgery, University of California San Francisco, San Francisco, CA, United States; ^2^Department of Neurology, University of California San Francisco, San Francisco, CA, United States

**Keywords:** DBS (deep brain stimulation), Parkinson’s disease, adaptive DBS, human neuroscience, sleep

## Abstract

Adaptive deep brain stimulation (aDBS) is a promising new technology with increasing use in experimental trials to treat a diverse array of indications such as movement disorders (Parkinson’s disease, essential tremor), psychiatric disorders (depression, OCD), chronic pain and epilepsy. In many aDBS trials, a neural biomarker of interest is compared with a predefined threshold and stimulation amplitude is adjusted accordingly. Across indications and implant locations, potential biomarkers are greatly influenced by sleep. Successful chronic embedded adaptive detectors must incorporate a strategy to account for sleep, to avoid unwanted or unexpected algorithm behavior. Here, we show a dual algorithm design with two independent detectors, one used to track sleep state (wake/sleep) and the other used to track parkinsonian motor state (medication-induced fluctuations). Across six hemispheres (four patients) and 47 days, our detector successfully transitioned to sleep mode while patients were sleeping, and resumed motor state tracking when patients were awake. Designing “sleep aware” aDBS algorithms may prove crucial for deployment of clinically effective fully embedded aDBS algorithms.

## Introduction

Commercially available sensing enabled deep brain stimulation (DBS) devices are now being used to treat epilepsy [Neuropace RNS, (Nair and Morrell, 2019)], movement disorders and obsessive-compulsive disorder (Medtronic Percept), (Feldmann et al., 2021; Frank et al., 2021; Jimenez-Shahed, 2021; Thenaisie et al., 2021). These devices typically record field potentials either cortically or subcortically and are designed to deliver personalized stimulation in response to sensed brain activity (Gunduz et al., 2019; Bronte-Stewart et al., 2020). For most indications, a neural biomarker of interest is typically selected and analysis is performed on predefined power bands in the frequency domain, computed from the sensed field potential recordings (Gunduz et al., 2019; Yin et al., 2021). Biomarkers are then compared to a predefined threshold and stimulation current is adjusted between stimulation amplitude limits. These adaptive algorithms are now routinely tested not only in clinical settings but at home (Gilron et al., 2021).

As studies transition from brief in clinic testing to chronic testing at home, it is important to consider that sleep has a profound influence on most biomarkers of interest. Sleep is typically disturbed in neurological conditions and could be modulated for therapeutic

purposes (Chen et al., 2019). However, to date, the “mental model” for adaptive DBS algorithms have not yet incorporated a special “mode” to detect sleep (Little and Brown, 2020). Sleep aware algorithms are important since control signals can cause unexpected or unwanted algorithm behavior in response to sleep related changes in brain physiology (Urrestarazu et al., 2009; Zahed et al., 2021).

**FIGURE 1:**
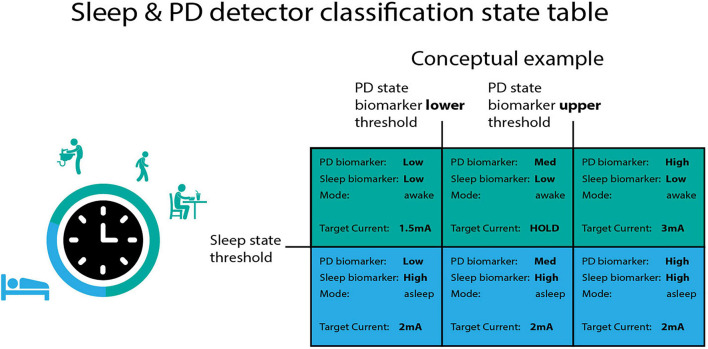
Independent classification of sleep and PD motor state. The summit RC + S can configure two independent linear detectors. These detectors operate using a “state table.” The control signal for the PD biomarker (which operates along the columns) has two thresholds, whereas the control signal for the sleep state has one threshold. Each quadrant in the state table can be defined with a unique target current in order to functionally separate sleep from wake “modes” in the adaptive DBS algorithm. In the conceptual example above, when the sleep control signal is above threshold target current ramps to 2 mA regardless of the PD state biomarker position with respect to PD state thresholds.

Here, we show a general purpose strategy to Figure 1 detect and respond to sleep using a dedicated “sleep detector” working in conjunction with an independent detector of specific motor signs in Parkinson’s disease (PD). The incorporation of sleep aware behavior into adaptive DBS (aDBS) algorithms may prove crucial to long term use of adaptive DBS protocols (Toth et al., 2020).

## Materials and Methods

### Participants and Device

**FIGURE 2:**
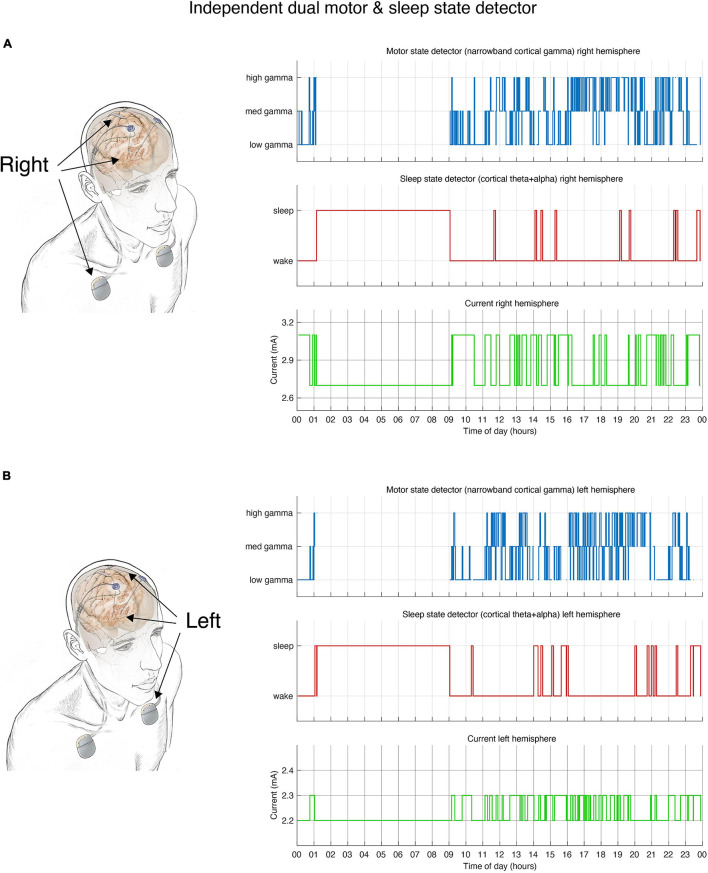
Dual independent PD and sleep state detectors. Detector operates in embedded fashion in each hemisphere independently. **(A)** Schematic drawing of lead locations indicating right hemisphere cortical sensing paddle; right STN stimulation lead and implanted pulse generator (IPG). Detector activity depicted in top 3 plots. **(B)** Schematic of the left hemisphere mirrors the right, bottom 3 plots show detector activity. **(A,B)** During hours in which the patient is awake the detector control signal (blue line) tracks parkinsonian motor signs using a narrowband gamma cortical signal (thought to indicate a pro-dyskinetic state). In this case stimulation cycles between 2.6 mA (if high levels of gamma detected) and 3.2 mA (low levels of gamma) in the right hemisphere (2.2–2.4 mA on the left). If sleep is detected (red line) by cortical alpha + theta control signal, stimulation is held at 2.6 mA (right) or 2.2 mA (left). Current is shown in green line. Time of day (24 h clock) is on *x*-axis.

Details of surgery, DBS implant, lead locations and device characteristics are extensively described in a prior publication (Gilron et al., 2021). Briefly, four individuals with PD referred for DBS were implanted bilaterally with cylindrical DBS leads in the subthalamic nucleus (STN) (Medtronic model 3389) and paddle-type quadripolar leads in the subdural space over MC (motor cortex, Medtronic model 0913025). The cortical and subcortical leads from each side were connected to an investigational sensing RC + S implantable pulse generator (IPG) allowing independent control of each hemisphere (Figure 2).

The Summit RC + S device is a rechargeable IPG that is capable of streaming data to a host computer from up to four field potential bipolar recordings (Stanslaski et al., 2018). Spectral power is computed within the device from (up to eight) predefined power bands. Power bands can be summed and input into two independent linear detectors that execute stimulation commands according to a state table (Figure 1). Data were imported from the RC + S raw format using a newly released package for RC + S analysis (Sellers et al., 2021).

### Deep Brain Stimulation Mental Model

**FIGURE 3:**
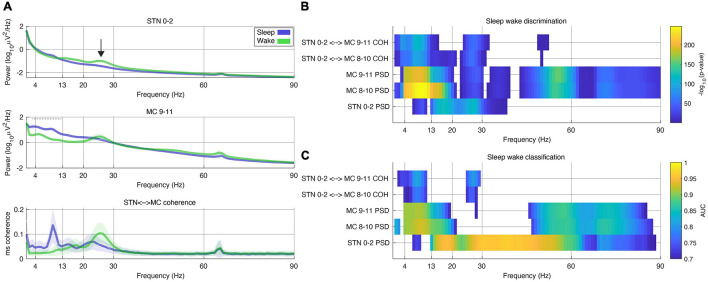
Frequencies discriminating between sleep and wake states during a period of 24 h in a single subject. **(A)** Power spectral density (PSD) plots of sleep and wake hours. These PSD data were computed from 30 s segments of field potentials classified by patient motor diary. Stimulation was held constant. Top plot – field potential from STN (subthalamic nucleus) contacts 0–2 (stimulation was constant at 2.7 mA on contact C + 1–). Middle plot – MC (motor cortex) contact 9–11. Bottom plot – magnitude squared (ms) coherence. In all plots shaded bars represent 0.5*standard deviation of segments. Black arrow (top plot) represents beta band activity that is commonly used as a marker of PD medication state and potential biomarker. Note decrease in beta band activity during sleep. Dashed horizontal gray bay in middle plot (MC 9–11) represents band used for sleep classification in this patient (Table 1). **(B)** Color plot of -log10 of individual *p*-values computed from *t*-tests across all bipolar recording contacts (higher values represent smaller *p*-values). Only frequency bins in which significant differences were found between sleep or wake states are displayed (the equivalent of a Bonferroni corrected value of *p* = 0.05 on this scale is 4). Each row represents PSDs (power spectral density) from motor cortex (MC), STN (subthalamic nucleus) or ms-coherence between the two. **(C)** Same data used in **(B)** was used to assess the out-of-sample classification with the area under the curve (AUC) computed using the ROC (receiver operating characteristic) curve. Plot was thresholded at an AUC of 0.7 (only higher scores shown).

One of the main challenges deploying adaptive algorithms chronically in the home environment is that sleep has a profound effect on cortical and subcortical field potentials. For instance, many algorithms for adaptive DBS in PD rely on responding to subcortical beta (12–30 Hz) as a control signal delivering less stimulation when subcortical beta is low. However, this may lead to inadequate stimulation for many patients since subcortical beta is also depressed during sleep (Figure 3), and some rely on stimulation to improve sleep (for example, to roll over in bed). Therefore there is a need to create a “sleep” aware algorithm that may switch modes when sleep is detected. To do so, we developed such an algorithm using the embedded “state table” in the RC + S IPG.

**TABLE 1:** Example of dual detector parameter set from a single patient and hemisphere.

**Setting**	**Value**	**Notes**
Stimulation rate	130.2 Hz	Stimulation rate
Stimulation pulse	60 μs	Stim pulse width
Stimulation contact	+1-case	Monopolar stimulation
**Shared parameters for both detectors**		
Time domain sampling rate	500 Hz	Sampling rate for time domain data
LPF1	450 Hz	Embedded low pass filter before amplification
LPF2	1,700 Hz	Embedded low pass filter after amplification
FFT interval	500 ms	Interval in which FFT is computed
FFT size	1,024	Number of points for onboard FFT computation
Ramp up rate	0.03 mA/sec	Rate at which stimulation changes from lower to higher amplitudes
Ramp down rate	0.12 mA/sec	Rate at which stimulation changes from higher to lower amplitudes
**Motor detector**		
Sense channel	+ 9−8	MC (motor cortex)
Linear detector power band input	64.45 – 66.41 Hz	Predefined power band for embedded on board power computation
Update rate	60	Number of FFT intervals averaged in non-moving average, represents 30 s of data. Defines algorithm rate (e.g., algorithm state is determined every 30 s). This is referred to as “detector count” below.
Onset	0	Number of detector counts must be **above** threshold to change to state. A value of 0 means that as soon as the threshold is crossed stimulation ramps to the target state.
Termination	4	Number of detector counts must be **below** threshold to change to state. Detector value must be **below** threshold for 2 consecutive minutes (update rate × 4; = 4 min in this case) before it transitions to state.
State change blank	30	In units of FFT interval. On state change power values are not computed into linear detector for 15 s (FFT interval × 30).
Target current state 0	3.1 mA	Target stim to ramp to when stimulation is below lower threshold (for the **first** linear detector- “LD0”)
Target current state 1	HOLD	Target stim to ramp to when stimulation between upper and lower thresholds (for the **first** linear detector – “LD0”)
Target current state 2	2.7 mA	Target stim to ramp to when stimulation is above lower threshold (for the **first** linear detector – “LD0”)
**Sleep Detector:**		
Sense channel	+9−8	MC (motor cortex)
Linear detector power band input	3.42 – 12.21 Hz	Predefined power band for embedded on board power computation
Update rate	60	Number of FFT intervals averaged in non-moving average, represents 30 s of data. Defines algorithm rate (e.g., algorithm state is determined every 30 s)
Onset	0	Number of detector counts must be above threshold to change to state (in “update rate” units).
Termination	10	Number of detector counts must be below threshold to change to state. Detector value must be below threshold for 5 consecutive minutes (update rate × 10; = 10 min in this case) before it transitions to state.
State change blank	30	In units of FFT interval. On state change power values are not computed into linear detector for 15 s (FFT interval × 30).
Target current state 3	2.7 mA	Target stim to ramp to when stimulation is below lower threshold (for the second linear detector – “LD1”)
Target current state 4	2.7 mA	Target stim to ramp to when stimulation between upper and lower thresholds (for the second linear detector – “LD1”)
Target current state 5	2.7 mA	Target stim to ramp to when stimulation is above lower threshold (for the second linear detector – “LD1”)

The “state table” (Figure 1) adaptive controller in the RC + S IPG was used to create two independent embedded adaptive detectors. The first linear detector tracked Parkinsonian motor state and the second tracked sleep state. The mental model for the first linear detector relied on following medication state as measured by biomarkers of Parkisonian “off” states (indicating low mobility) and “on” states (indicating potential for dyskinesia). During off periods patients received additional stimulation and in on states they received less stimulation in order to avoid stimulation induced dyskinesia (Gilron et al., 2021). The second linear detector relies on tracking sleep state as measured by a sleep biomarker. When sleep was detected, constant stimulation was delivered according to the state table, regardless of the position of the first linear detector (equivalent to clinically optimized open loop stimulation). Figure 1 contains a sample state table.

Of note, RC + S has two independent programmable linear detectors. Each detector has two thresholds, which result in 9 possible unique states. Each state can be programmed with a specific target amplitude for each program (and specific rate for each state across programs). Here we are only using 6 of the possible 9 states, but future studies may use all 9 states (for example, for detection of multiple motor signs, or specific sleep stages). States are numbered (0–8) starting top left and ending bottom right.

### Detector Settings

Biomarkers for Parkinsonian “on” and “off” states and sleep states (wake/sleep) were selected empirically per patient (Gilron et al., 2021). First, frequency bands in which oscillations (local maxima in the field potential power spectral density) were present in either STN or cortical field potentials, were pre-selected and configured using the embedded power detectors. Next, each patient streamed “training” data during their activities of daily living across several “wake/sleep” cycles and Parkinsonian “on/off” cycles. Patient state was assessed using a motor diary, wearables and patient self report. Biomarkers that best seperated Parkinsonian state and sleep state were empirically selected. Detector thresholds were initially set at 25 and 75% percentiles of the range of training data for embedded power detector for Parkinsonian state and 50% percentile for sleep state detector and empirically adjusted over several days until satisfactory performance was achieved. Table 1 contains the complete parameter set used to program the detector in one patient during a single 24 h period.

Rapid eye movement (REM) periods could cause the sleep detector to mistakenly identify a “wake” state. In order to counter “wake” classification in these instances we used long termination rates such that the biomarker of interest must be below the threshold for a certain amount of time before it transitions out of the sleep state. Most of the algorithm adjustments in patient 1 (throughout the testing period) involved gradually shortening the termination value at the expense of some misclassification during putative REM sleep. The detector was initially designed for increased sensitivity at the cost of decreased specificity. The sensitivity vs. specificity tradeoff may be an important design consideration for future detectors.

### Data Collection for Testing Period

**FIGURE 4:**
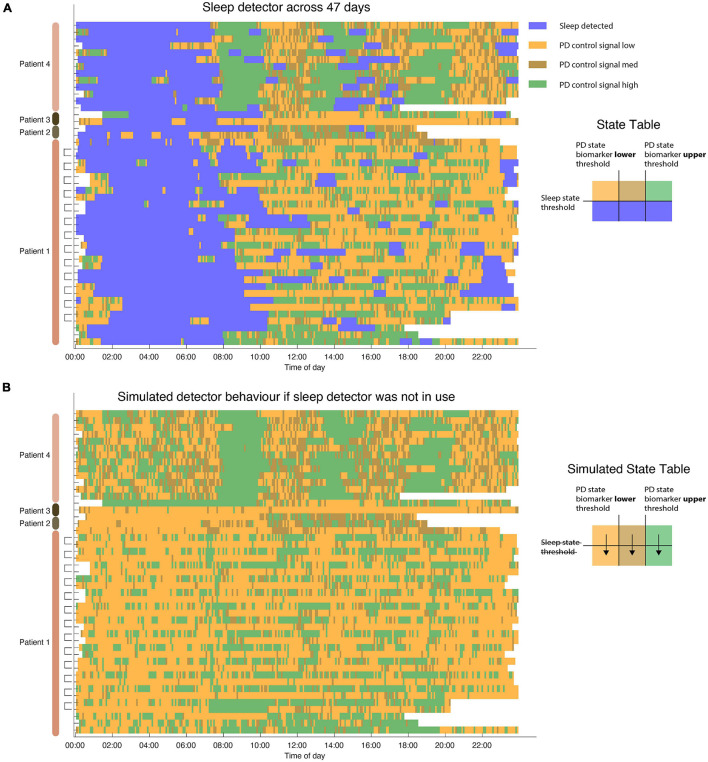
Sleep detector performance across 47 days of sleep. **(A)** Dual detector states across 4 patients in 6 hemispheres. Each row represents a single 24 h period. Note that in patient 1 each hemisphere was running independently (measuring sleep states using L/R hemispheres, respectively). Detector traces from the same day (coming from two different pulse generators) are indicated in brackets. Sleep is not only identified during night hours but also occurs in some patients during daytime naps. **(B)** Simulated state table showcase PD state detector state if sleep detector was not in place during same hours. Without relying on a dedicated sleep detector PD state control signal still undergoes many state changes during sleep hours. Blank (white) periods indicate moments in which the detector was not active or data do not exist.

Though the RC + S device is capable of streaming a rich array of information including time domain field potentials and adaptive state, it can also be programmed to only store a log of the adaptive DBS state (according to the state table). This log is stored in a FIFO (first in first out) buffer which can be downloaded on demand. Patients collected DBS state data during long term tests of embedded adaptive detectors in which the embedded detector was deployed for up to a week at a time. During this time patients used custom software^1^ to download these logs from the device on a daily basis. This had the advantage of allowing the patient full mobility without the need to be near a computer for wireless streaming. In addition, it avoided losing data due to dropped packets (Sellers et al., 2021). Data were tested over the course of 47 days (24 h) across four patients and six hemispheres. Patient one used sleep classifiers for 17 days but frequently ran a classifier concurrently in both hemispheres, generating 30 data sets (Figures 2, 4), patient 2 ran a classifier for 2 days in one hemisphere, patient 3 ran a classifier for 2 days, 1 day for each hemisphere, and patient 4 ran a classifier for 13 days in one hemisphere.

### Evaluation of Sleep Detector Performance

The performance of the sleep detector was checked empirically in each patient before it was deployed. Using patient motor diaries, wearables and the RC + S onboard accelerometer, patient sleep state was verified and a concordance between sleep state and the objective (actigraphy based) and subjective (motor diary) measures was computed.

In 27/47 nights of sleep we collected motor diaries and wearable actigraphy data. In filling out motor diaries patients estimated their sleep state in 30 min increments. Concurrently a wearable watch (PKG, Global Kinetics) was worn by patients during testing (Joshi et al., 2019). The PKG produced a report that estimated a median bradykinesia value every 30 min. A value above “80” in the bradykinesia metric from the PKG watch was interpreted as “asleep” (Gilron et al., 2021).

To test the statistical performance of the classifier we computed a non-parametric *p*-value for each 24 h period using the following procedure: We compared the concordance between the output of the sleep detector and the “true” patient state as indicated by either the motor diary or the PKG watch. To test the statistical significance of this concordance (accuracy) value we created a non-parametric null distribution of classification (a random choice between sleep/wake classification at each point in time repeated 10,000 times for each 24 h period). The concordance result of the embedded adaptive state classification was then compared to the null distribution of concordances to derive a *p*-value. All *p*-values were corrected for multiple comparisons using the Bonferroni method. In addition the sensitivity and specificity of the sleep classification was computed for each 24 h period.

### Effect of Sleep State on Power and Coherence Metrics

In order to assess the effect of sleep on biomarker power and coherence metrics we had one patient stream neural data (in addition to classifier state) during a 24 h period when the sleep detector was active, but stimulation was held constant in the clinically optimized current settings (2.7 mA, C + 1−, 60 μs, 130.2 Hz). Sleep states were defined by the classifier and PSD (power spectral density) and coherence metrics were computed from bipolar recordings in STN and MC (motor cortex) using 30 s segments of continuous data [as described in Gilron et al. (2021)].

To assess the response of other frequencies on the discrimination of sleep state (wake/asleep, as defined by patient motor diaries) frequencies were swept in 1 Hz increments using PSD and coherence metrics with a 2 Hz sliding window. The data from each frequency bin was subjected to a two sided *t*-test and computed for each frequency, contact pair and measure (coherence/psd). *P*-values were corrected for multiple comparisons using the Bonferroni method.

Since our embedded detector used cortical alpha and theta bands as the sleep detector, we wanted to evaluate the capacity of subcortical and coherence based metrics to classify sleep state as well. Using the cortically based detector labels as ground truth, we calculated the mean AUC using a 5-fold stratified cross validated linear discriminant model across all frequency bands. This allowed us to assess the potential performance of the detector using subcortical sensing that is more readily available in commercial devices as well as using coherence based metrics.

## Results

Sleep state was reliably captured by a cortical biomarker as verified using patient motor diaries and self report. Each IPG (implanted pulse generator) controlled stimulation to one hemisphere with an embedded detector for sleep and an additional detector tracking PD state (Figure 2). The mean concordance between sleep measurements across both hemispheres (in cases in which dual detectors were deployed) was 88% (range 77–98%) across thirteen 24-h periods.

In one subject the embedded detector was run over a period of 24 h while streaming neural data to a research computer [as described in Gilron et al. (2021)]. This was done in order to examine other frequency bands and brain recording locations that might also dissociate sleep from wake states. In particular there is interest in subcortical classification of sleep states, as cortical sensing is not currently offered outside of IDE (investigational device exemption) studies. Subcortical alpha, beta and low gamma all significantly dissociated sleep from wake states (Figure 3). Cortical theta, alpha, beta and broadband gamma discriminate sleep states as well. Finally coherence between subcortical and cortical structures was able to dissociate sleep states in the theta, alpha and beta bands.

In addition to investigating other frequencies that can distinguish sleep from wake states we also assessed the out of sample classification of sleep states across the same 24 h period. Using 5-fold cross validation we found that STN alpha, beta and gamma all discriminate sleep states with peak AUC scores of 0.7 (using the cortical theta-alpha detector as “ground truth”).

Algorithm performance was tested using embedded mode during which patients are not tethered to a computer and the adaptive algorithm operates in embedded mode in real-time. Across 47 days (4 unique patients, 6 hemispheres) algorithm captured sleep state (Figure 4A). Algorithm performance was stable across months (maximum span between recording was 4 months). To assess whether a dedicated detector for sleep was needed we simulated algorithm performance without the use of a sleep detector. Indeed, all patients displayed large variation in the control signal during periods of sleep (Figure 4B) which could result in unwanted behavior depending on the aDBS (adaptive DBS) algorithm used to control PD motor states during waking hours. In some cases this would result in lower stimulation levels during sleep which could produce adverse effects on sleep in PD patients (Zahed et al., 2021).

**FIGURE 5:**
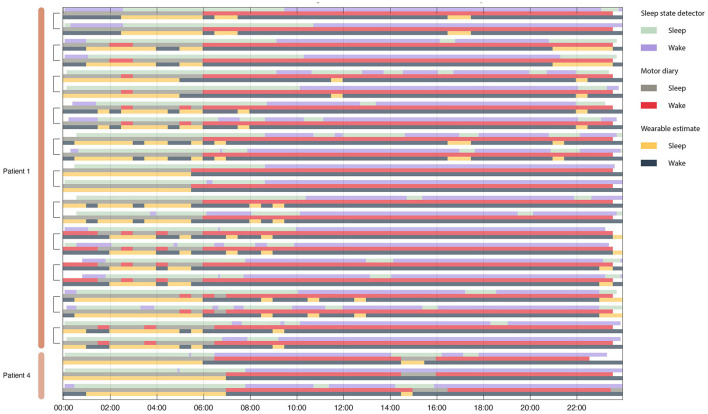
Concordance between motor diary, wearable watch and sleep state detector. Rows are organized in triplicate such that each triad represents a single 24 h period from one patient. The top row in the triplicate represents the output of the embedded sleep detector. The control signals for the embedded sleep detector were cortical field potentials in the 3–12 Hz range. This detector operated in real time during activities of daily living and in the absence of any temporal information. The middle row represents (subjective) patient motor diary information in which the patient indicated his sleep/wake state in 30 min increments. Finally, the bottom row indicates wearable estimates of patient state (sleep wake). Brackets indicate recordings from the same day from two independent implanted pulse generators – one in each patient hemisphere.

To validate the performance of the sleep detector objective and subjective metrics of sleep state were collected. Patients filled out motor diaries (subjective) and wore a wearable watch (objective), both of which produced estimates patient sleep state that could be used to assess classifier performance (Figure 5).

**FIGURE 6:**
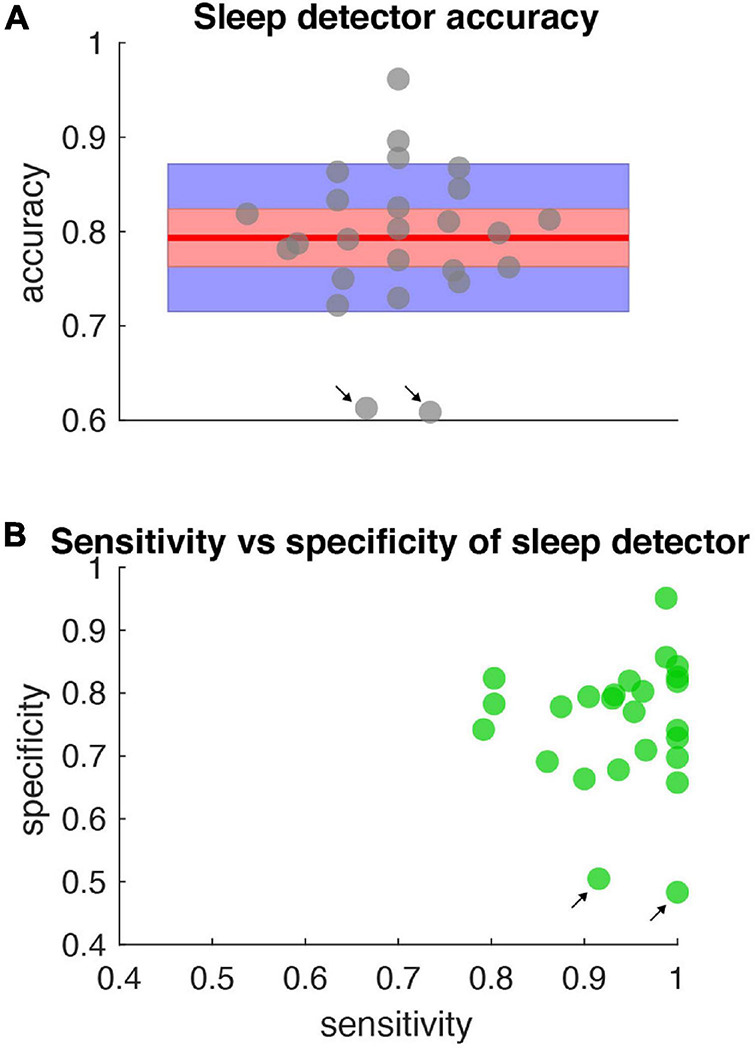
Accuracy, specificity, sensitivity of sleep detector. **(A)** Sleep detector accuracy (as measured by objective metric – PKG watch) for all data shown in Figure 5. Gray dots represent accuracy scores (jittered along *x*-axis for clarity). Blue shading represents one standard deviation and red shading represents the 95% confidence interval. Red horizontal line is the mean (0.79). All accuracy metrics were significant compared to a null distribution of classifier performance and corrected for multiple comparisons. **(B)** Specificity and sensitivity metrics for the same data represented in **(A)**. Black arrows indicate days in which high sensitivity but low specificity was present.

All sleep night detections were significantly above chance (the null distribution had concordances between 38 and 60% with a mean at 50%) for both motor diary and wearable “ground truth” metrics (Figure 6).

## Discussion

This study showcases a method to incorporate sleep sensing into adaptive DBS algorithms. In Summit RC + S we deployed two embedded linear detectors that operate independently. Tracking sleep states using embedded detectors is stable and repeatable, shown here across 47 days of embedded detector performance in four patients (Figure 4). During these days the algorithm successfully transitioned to a predefined “sleep mode” when patients were asleep, delivering targeted constant stimulation, whereas during waking hours a PD state tracking mode was entered in which the adaptive algorithm tracked PD related motor state changes (Gilron et al., 2021).

The sleep detector algorithm achieved high concordance with external measurements of sleep as measured by both objective (motor diary) and subjective (watch) metrics. The high concordance is notable since the detector classified sleep solely based on field potential information in real time, during activities of daily living without access to actigraphy or temporal based information (Figure 5). The sleep detector achieved high degrees of sensitivity and specificity but future sleep algorithms may explore sensitivity and specificity trade offs depending on the application (Figure 6).

Sleep has dramatic effects on cortical and subcortical field potentials (Gilron et al., 2021; Zahed et al., 2021). This includes broadband reductions in gamma frequency as well as increases in alpha frequencies and reductions in canonical parkinsonian oscillatory activity in beta frequency. Since beta frequencies are a common target for adaptive DBS studies in PD, addressing sleep induced reductions may be critical for future algorithm development.

Though our sleep study used cortical alpha and theta band activity to classify sleep state we show that other bands, notably subcortical bands achieve high classification rates (larger than AUC of 0.9 – Figure 3C). This high degree of sensitivity and specificity from a variety of power bands, target brain locations and coherent network activity suggest that a variety of control algorithms might be employed to incorporate sleep into adaptive DBS algorithms.

Using a separate independent detector for sleep allows adaptive DBS algorithms to finetune their response to diurnal fluctuations in control signals that may operate on different time scales than sleep changes. For example, targeting beta bursts has long been proposed as a mechanism to target pathological beta burst oscillations in PD (Little and Brown, 2012, 2020; Tinkhauser et al., 2017; Velisar et al., 2019; Bronte-Stewart et al., 2020; Petrucci et al., 2020), but these bursts operate on the timescale of 200–800 milliseconds whereas sleep related field potential changes take place on timescales of minutes to hours. The use of independent detectors allows each detector to operate using disperate timescales most appropriate for capturing desired state.

Sleep was frequently detected during the day, corresponding to daytime naps (Figure 5). This highlights a benefit of using physiological measures of sleep state rather than simpler chronologically scheduled implant schedules (Toth et al., 2020). Other potential methods for sleep classification are actigraphy based (to determine position and activity level) or simple patient control (patient switching to a sleep “mode”). Appropriately targeting and incorporating sleep into aDBS algorithms has benefits for risk mitigation, as it does not rely on the patient to remember to switch device state or actigraphy methods which may produce false negatives (such as lying in bed while awake).

Sleep aware aDBS may also aid in algorithm development as it allows testing putative algorithm performance (Figure 4B). Prior to implementing adaptive detectors it is useful to test algorithm performance by examining state changes without stimulation amplitude changes. By incorporating a dual detector strategy that incorporates sleep control signals as well as dedicated detectors tracking patient state one can separate sleep effects from other effects on the control signal. This can allow *a priori* testing to help avoid unwanted or unexpected algorithm performance during sleep. This could prove particularly useful for adaptive algorithms for which operation during sleep is important.

Future studies may attempt to more selectively target sleep by deploying personalized therapy depending on patient sleep state (for example, REM sleep versus deep sleep). Sleep itself is often disturbed in patients with DBS due to their underlying neurological conditions, and this represents a major non-motor contributor to quality of life. Adaptive strategies that improve sleep quality may work in tandem with daytime strategies to address non-motor as well as motor dysfunction in PD.

## Data Availability Statement

The raw data supporting the conclusions of this article will be made available by the authors, without undue reservation.

## Ethics Statement

The studies involving human participants were reviewed and approved by the University of California, San Francisco, Institutional Review Board (IRB) under a physician-sponsored investigational device exemption (IDE) from the FDA (protocol G180097). The patients/participants provided their written informed consent to participate in this study.

## Author Contributions

RG and PS conceived the study and experiments. SL provided clinical supervision. RP wrote the software interface for Summit RC + S. RG and RW collected the data. RG and SL provided key analytic tools. RG drafted the manuscript and figures. All authors contributed to manuscript revision, read, and approved the submitted version.

## Conflict of Interest

Devices were provided at no charge by Medtronic. PS is inventor on US patent 9,295,838 “Methods and systems for treating neurological movement disorders”; the patent covers cortical detection of physiological biomarkers in movement disorders, which is also discussed in this manuscript. The remaining authors declare that the research was conducted in the absence of any commercial or financial relationships that could be construed as a potential conflict of interest.

## Publisher’s Note

All claims expressed in this article are solely those of the authors and do not necessarily represent those of their affiliated organizations, or those of the publisher, the editors and the reviewers. Any product that may be evaluated in this article, or claim that may be made by its manufacturer, is not guaranteed or endorsed by the publisher.

## Footnotes

1
https://github.com/openmind-consortium/App-SCBS-PatientFacingApp
